# Efficacy and safety of treatments in newly diagnosed adult primary immune thrombocytopenia: A systematic review and network meta-analysis

**DOI:** 10.1016/j.eclinm.2022.101777

**Published:** 2022-12-14

**Authors:** Yun Wang, Lei Sheng, Fengjiao Han, Qiuyu Guo, Zihan Zhang, Yu Hou, Qi Feng, Hai Zhou, Xuebin Ji, Jun Peng, Ming Hou, Miao Xu

**Affiliations:** a Department of Hematology, Cheeloo College of Medicine, Qilu Hospital of Shandong University, Jinan, China; b Department of General Surgery, Cheeloo College of Medicine, Qilu Hospital of Shandong University, Jinan, China; c Advanced Medical Research Institute, Shandong University, Jinan, China

**Keywords:** Primary immune thrombocytopenia, Network meta-analysis, Randomized controlled trials, Dexamethasone, Prednis(ol)one, Rituximab, rhTPO, All-trans retinoic acid, Oseltamivir, Tacrolimus, ITP, primary immune thrombocytopenia, NMA, network meta-analysis, RCTs, randomised controlled trials, DEX, dexamethasone, PRD, prednis(ol)one, RTX, rituximab, RA, all-trans retinoic acid, OSE, oseltamivir, TAC, tacrolimus, OR, odds ratio, IVIg, intravenous immunoglobulin, ASH, American Society of Hematology, TPO-RAs, thrombopoietin receptor agonists, AEs, adverse events, CrI, credible intervals, CI, confidence interval, SUCRA, the surface under the cumulative ranking curve, QoL, quality of life, Anti-D, Rho(D) immune globulin, rhTPO, recombinant human thrombopoietin, mPRD, methylprednisolone

## Abstract

**Background:**

Immune thrombocytopenia is an autoimmune disease characterised by decreased platelet count. In recent years, novel therapeutic regimens have been investigated in randomised controlled trials (RCTs). We aimed to compare the efficacy and safety of different treatments in newly diagnosed adult primary immune thrombocytopenia.

**Methods:**

We did a systematic review and network meta-analysis of RCTs involving treatments for newly diagnosed primary immune thrombocytopenia. PubMed, Embase, the Cochrane Central Register of Controlled Trials, and ClinicalTrials.gov databases were searched up to April 31, 2022. The primary outcomes were 6-month sustained response and early response. Secondary outcome was grade 3 or higher adverse events. This study is registered with PROSPERO (CRD42022296179).

**Findings:**

Eighteen RCTs (n = 1944) were included in this study. Pairwise meta-analysis showed that the percentage of patients achieving early response was higher in the dexamethasone-containing doublet group than in the dexamethasone group (79.7% *vs* 68.7%, odds ratio [OR] 1.82, 95% CI 1.10–3.02). The difference was more profound for sustained response (60.5% *vs* 37.4%, OR 2.57, 95% CI 1.95–3.40). Network meta-analysis showed that dexamethasone plus recombinant human thrombopoietin ranked first for early response, followed by dexamethasone plus oseltamivir or tacrolimus. Rituximab plus prednisolone achieved highest sustained response, followed by dexamethasone plus all-trans retinoic acid or rituximab. Rituximab plus dexamethasone showed 15.3% of grade 3 or higher adverse events, followed by prednis(ol)one (4.8%) and all-trans retinoic acid plus dexamethasone (4.7%).

**Interpretation:**

Our findings suggested that compared with monotherapy dexamethasone or prednis(ol)one, the combined regimens had better early and sustained responses. rhTPO plus dexamethasone ranked top in early response, while rituximab plus corticosteroids obtained the best sustained response, but with more adverse events. Adding oseltamivir, all-trans retinoic acid or tacrolimus to dexamethasone reached equally encouraging sustained response, without compromising safety profile. Although this network meta-analysis compared all the therapeutic regimens up to date, more head-to-head RCTs with larger sample size are warranted to make direct comparison among these strategies.

**Funding:**

10.13039/501100001809National Natural Science Foundation of China, Major Research Plan of 10.13039/501100001809National Natural Science Foundation of China, 10.13039/501100007129Shandong Provincial Natural Science Foundation and Young 10.13039/100012620Taishan Scholar Foundation of Shandong Province.

**Research in context**

**Evidence before this study**

Primary immune thrombocytopenia (ITP) is an autoimmune-mediated haematological disorder characterised by decreased platelet count and the increased tendency of bleeding. Broad search terms were used (‘meta-analysis’, ‘immune thrombocytopenia, and ‘newly diagnosed’) to search PubMed, Embase, Cochrane Library, and ClinicalTrials.gov databases up to April 31, 2022 and 4 pairwise meta-analyses were identified, comparing low-dose IVIg *vs* high-dose IVIg, dexamethasone *vs* predis(ol)one, rituximab plus dexamethasone *vs* dexamethasone monotherapy, respectively. However, there is no underway systematic review and network meta-analysis to compare therapeutic strategies emerged in recent years.

**Added value of this study**

This study reviews all the up-to-date treatments for newly diagnosed adult primary ITP from RCTs with medium to high quality, including dexamethasone, prednis(ol)one, all-trans retinoic acid plus dexamethasone, oseltamivir plus dexamethasone, rituximab plus dexamethasone, tacrolimus plus dexamethasone, IVIg plus prednisone, rhTPO plus dexamethasone, methylprednisolone with or without prednisone, low-dose prednisone, rituximab with prednisolone. This work updates the existing evidence of meta-analyses assessing the efficacy and safety of 11 different regimens for newly diagnosed ITP.

**Implications of all the available evidence**

All the combined regimens demonstrated higher remission rate for newly diagnosed ITP than corticosteroids alone in both early and sustained response. rhTPO plus dexamethasone ranked top in early response, while rituximab plus corticosteroids obtained the best sustained response. Three brand-new regimens (dexamethasone plus all-trans retinoic acid, oseltamivir, or tacrolimus) showed satisfactory response with relatively low incidence of adverse events, however, only one trial was performed respectively.

## Introduction

Immune thrombocytopenia (ITP) is an acquired autoimmune disease characterised by decreased platelet count and increased tendency of bleeding, owing to excessive platelet destruction and/or insufficient platelet production.^1^, ^2^, ^3^ The incidence of ITP is 3.3/100,000 adults per year with a prevalence of 9.5 per 100,000 adults.^4^ Majority of patients are asymptomatic or experience only mild mucocutaneous haemorrhage, while about 5% of patients may suffer from lethal bleeding, such as intracranial hemorrhage.^5^

Since the first International Consensus Report of ITP released in 2008, corticosteroid, intravenous immunoglobulin (IVIg), and Rho(D) immune globulin (Anti-D) have been listed as the first-line treatments.^5^^,^^6^ As the initial choice and most frequently used therapy, corticosteroids are recommended by American Society of Hematology (ASH) for adults with newly diagnosed ITP (<3 months) and a platelet count of <30 × 10^9^/L, whether bleeding or not.^6^ Approximately 80% of patients with ITP respond to the corticosteroids, but over 50% relapse after the steroids taper off.^7^^,^^8^ Over the last two decades, there are numerous investigations on the pathogenesis, further emphasizing the heterogeneity and complication of ITP. In recent years, several novel drugs, rituximab and thrombopoietin receptor agonists (TPO-RAs), such as romiplostim, eltrombopag, avatrombopag, have been developed and applied in the management of ITP.^6^ Moreover, scientists and clinicians repurposed some readily available drugs based on the updates on pathogenesis of ITP, such as all-trans retinoic acid, tacrolimus, oseltamivir and so on, in the management of ITP. Several recent randomised controlled trials (RCTs) demonstrated adding these drugs to corticosteroids monotherapy can provide extra benefits for patients with newly diagnosed ITP.^9^, ^10^, ^11^

However, confronted with multiple options, it is difficult to perform head-to-head RCTs between every two regimens to determine the optimal treatment. To address this question reasonably, we conducted a systematic review and network meta-analysis directly and indirectly to compare all the data from the RCTs and make pairwise comparisons.^12^ Here, we revealed the differences in efficacy and safety among all types therapeutic regimens and their ranking probabilities, providing evidence for the optimal treatment option in the newly diagnosed ITP.

## Methods

### Search strategy and selection criteria

This network meta-analysis was performed in accordance with the PRISMA extension statement. The research was registered with PROSPERO (CRD42022296179). PubMed, Embase, the Cochrane Central Register of Controlled Trials, and ClinicalTrials.gov databases were searched by two researchers independently to find relevant articles up to April 31, 2022. For multiple reported data of an outcome in the same trial, only the most recent data was kept. The detailed search strategy was shown in Appendix p 1. Then we screened the eligible studies by skimming through the titles and abstract of these articles. We included published phase II/III RCTs reported in English that met the following inclusion criteria: Trials that enrolled patients (age ≥ 18 years) with previously untreated, newly diagnosed (≤3 months), primary immune thrombocytopenia who had platelet count of less than 30 × 10^9^/L or a platelet count of less than 50 × 10^9^/L and clinically significant bleeding symptoms. To expand diversity of treatment options, this study includes latest conference abstract. We excluded trials with secondary or chronic or refractory or relapsed primary immune thrombocytopenia or with receiving previous treatments. Patients who are pregnant or under 18 years and articles not written in English were also excluded.

Two researchers extracted data independently. If they have a contradiction, they reach consensus by negotiation or seeking help for the senior researchers. Main data of qualified trials such as study ID, first author, publication year, number of patients, treatments and outcomes were extracted into a spreadsheet for further analysis. Two investigators independently extracted data parameters using a unified data extraction form.

### Data analysis

The primary outcomes were 6-month sustained response and early response. Secondary outcome was grade 3 or higher adverse events (≥3 AEs). All available direct and indirect evidence was synthesised to compare different treatments in terms of efficacy and safety, reported as odds ratio (OR) and corresponding 95% credible intervals (CrI).

Two investigators (Y.W. and L.S.) independently assessed risk of bias of individual studies. Any disagreement was discussed and resolved by M.X. and M.H. to reach a consensus. The bias risk of included trials was assessed as low, high, or unclear using the Cochrane Risk of Bias Tool with the following aspects: random sequence generation, allocation concealment, blinding of participants and personnel, blinding of outcome assessment, incomplete outcome data, selective outcome reporting and other sources of bias.

Network plots were generated to indicate direct or indirect comparison of different treatments using Stata (version 15.0). Network meta-analyses were performed in a Bayesian framework using a Markov Chain Monte Carlo simulation technique within the GEMTC package in the R-Statistics and the J.A.G.S. program as previously described.^13^ Fixed-effect models were used since in most cases the treatment of interest was evaluated in single trial.^14^ We used non-informative uniform and normal prior distributions to fit the model, with four different sets of initial values. For each outcome, 150,000 sample iterations were generated with 100,000 burn-ins and a thinning interval of 10. Model convergence (reaching a stable equilibrium distribution) was assessed using trace plots and the Brooks-Gelman-Rubin statistic (in Appendix pp 3–4). Once convergence was established, the posterior distributions for the model parameters were obtained.

Pairwise conventional meta-analyses were further performed with frequentist method for head-to-head trials. Heterogeneity was assessed between studies using the Q test and I^2^ statistic. The estimated I^2^ values under 25%, between 25% and 50%, or over 50% indicated low, moderate, or high heterogeneity, respectively. We produced a pooled odds ratio (OR) with its 95% confidence interval (CI) using a random effects model as a conservative estimate. Pooled OR using a fixed effects model was also performed to make a sensitive analysis, as well as to test the consistence with the estimate generated in network meta-analyses. Comparison-adjusted funnel plot was used to assess the presence of small-study effects or publication bias.^15^

Ranking probabilities were estimated for all treatments of being at each rank for each intervention. A treatment hierarchy was determined using the probability of being the best treatment by using the surface under the cumulative ranking curve (SUCRA; score of 0–1 and 1 is the best).

In addition to the principal analyses, sensitivity analysis was conducted to test the robustness and reliability of results by excluding one trial with about 30% of previously treated cases.

### Ethical statement

We used relevant data from public databases, including PubMed, Embase, the Cochrane Central Register of Controlled Trials, and ClinicalTrials.gov databases. Ethical approval and informed consent (if relevant) was covered in the original studies and was not applicable for this study.

### Role of the funding source

The funder of the study had no role in study design, data collection, data analysis, data interpretation, or writing of the report. The corresponding authors had full access to all the data in the study and had final responsibility for the decision to submit for publication.

## Result

**Fig. 1:**
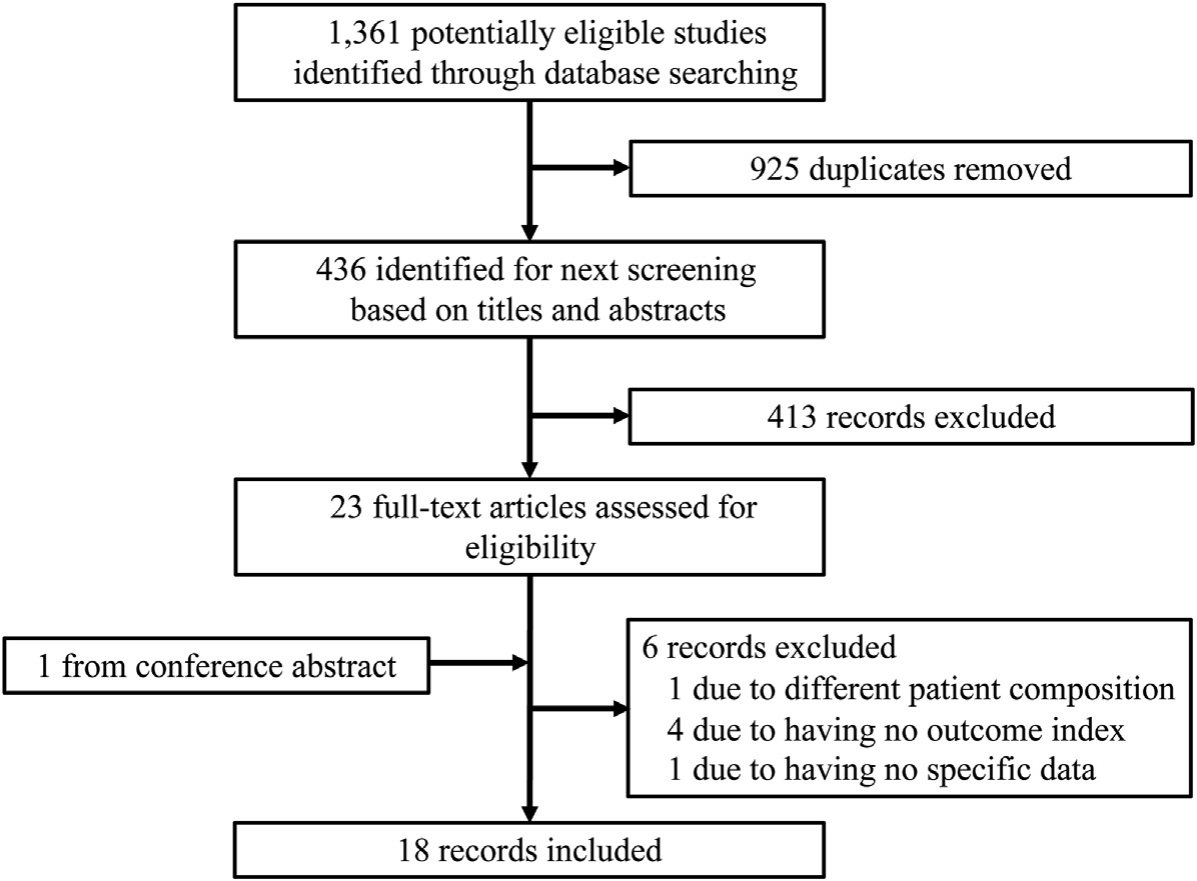
**Study selection flowchart**.

**Fig. 2:**
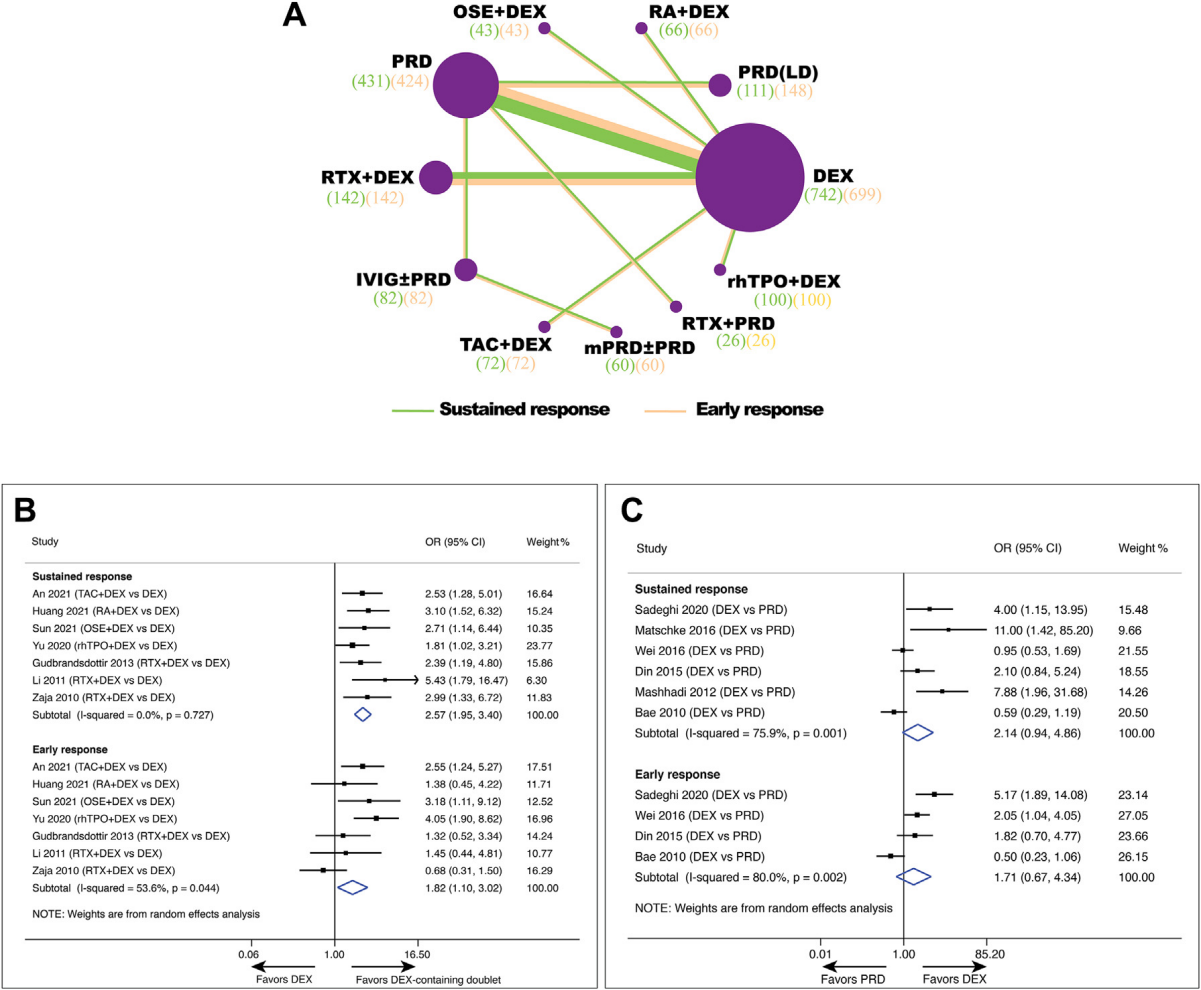
**Pairwise meta-analysis of comparison of different treatments on early response and sustained response in newly-diagnosed adult primary immune thrombocytopenia**. (A) Network diagrams of comparisons on early response (ER) and sustained response (SR). Each circular node represents a type of treatment. Each line represents a type of head-to-head comparison. Node size and line thickness are weighted according to the number of studies evaluating each treatment and direct comparison, respectively. The total number of patients receiving a treatment is shown in brackets. (B) Pooled odds ratio (OR) of sustained response and early response in comparison of dexamethasone-containing doublet versus dexamethasone. (C) Pooled odds ratio (OR) of sustained response and early response in comparison of dexamethasone versus prednisone. CI, confidence interval; DEX, dexamethasone; IVIG, intravenous gammaglobulin; mPRD, methylprednisolone; OR, odds ratio; OSE, oseltamivir; PRD, prednisone; RA, all-trans retinoic acid; rhTPO, recombinant human thrombopoietin; RTX, rituximab; TAC, tacrolimus.

According to database search, 1361 articles that were published in English from the inception to April 31, 2022 were retrieved. The concrete search flowchart is presented in Fig. 1. We include 11 therapeutic regimens in 18 articles, including dexamethasone, prednis(ol)one, all-trans retinoic acid plus dexamethasone, oseltamivir plus dexamethasone, rituximab plus dexamethasone, tacrolimus plus dexamethasone, IVIg plus prednisone, rhTPO plus dexamethasone, methylprednisolone with or without prednisone, low-dose prednisone, rituximab with prednisolone (Fig. 2A). Relevant information was extracted in the Table 1. One article was excluded as it belongs to conference abstract without concrete outcome data.^16^ The other study was excluded because of its different patient composition, in which the number of the patients with relapsed ITP accounted for over 45%.^17^ Four articles were excluded for no endpoints of interest.^18^, ^19^, ^20^, ^21^ The network is shown in Fig. 2A.

**Table 1:** Key features of included studies.

Study ID	Country	Number of participants (I/C)	Sex (M/F)	Age (y), median (I/C)	Baseline PLT count/median (×10^9^/L) (I/C)	Intervention	Regimen (I)	Comparison	Regimen (C)
Mazzucconi 1985	Italy	37/32	17/52	27/27	<60/<60	PRD	PRD 0.5 mg/kg × 1m	PRD	PRD 1.5 mg/kg × 1m
Bellucci 1988	France	111/112	66/157	40/43	24.8/24	PRD	PRD 0.25 mg/kg × 3w	PRD	PRD 1 mg/kg × 3
Jacobs 1994	South Africa	26/17	9/34	33/33	NM/NM	IVIG ± PRD	IVIG 400 mg/kg × 5d + PRD 1 mg/kg	PRD	PRD 1 mg/kg
Godeau 2002	France	56/60	39/77	40/37.5	<20/7	IVIG ± PRD	IVIG 0.7 mg/kg × 3d + PRD 1 mg/kg × 18d	mPRD ± PRD	mPRD 15 mg/kg × 3d + PRD 1 mg/kg × 18d
Praituan 2009	Thailand	18/18	8/28	44.9/39.5	8.5/10.3	DEX	DEX 40 mg × 4d	PRD	PRD 60 mg × 14d
Bae 2010	Korea	76/75	45/105	44/44	<30/<30	DEX	DEX 40 mg × 4d × 1–2	PRD	PRD 1 mg/kg × 28d
Zaja 2010	Italy	49/52	41/60	49/47	<20/<20	RTX + DEX	RTX 375 mg/m^2^ × 4w + DEX 40 mg × 4d	DEX	DEX 40 mg × 4d
Li 2011	China	31/31	25/37	26/24	7/6	RTX + DEX	RTX 100 mg × 4w + DEX 40 mg × 4d	DEX	DEX 40 mg × 4d
Mashhadi 2012	Iran	30/30	13/47	24.9/27.2	13.9/10.4	DEX + PRD	DEX 40 mg × 4d + PRD 1 mg/kg × 7d	PRD	PRD 1 mg/kg × 28d
Gudbrandsdotir 2013	Denmark	62/71	63/70	51/58	13/14	RTX + DEX	RTX 375 mg/m^2^ × 4w + DEX 40 mg × 4d	DEX	DEX 40 mg × 4d
Din 2015	China	61/29	40/50	30/29	<20/11	DEX	DEX 40 mg × 4d × 3	PRD	PRD 1 mg/kg × 4w
Wei 2016	China	95/97	56/136	43/44	7/8	DEX	DEX 40 mg × 4d × 1/2	PRD	PRD 1 mg/kg × 4w
Matschke 2016	Germany	13/9	13/9	46/43	5/2	DEX	PRD 1 mg/kg × 1w + DEX 0.6 mg/kg × 4d × 6	PRD	PRD 1 mg/kg × 2w
Datta 2018	India	26/26	NM	NM	NM	RTX + PRD	PRD 1 mg/kg × 4w + RTX 100 mg/w × 4w	PRD	PRD 1 mg/kg × 4w
Yu 2020	China	100/96	64/132	42/45	7/7	DEX + rhTPO	rhTPO 300 U/kg/d × 14d + DEX 40 mg/d × 4d	DEX	DEX 40 mg/d × 4d
Sadeghi 2020	Iran	36/36	NM	>18/>18	NM	DEX	DEX 40 mg × 4d × 3	PRD	PRD 1 mg/kg × 4w
Sun 2021	China	43/47	40/43	47/40	9/11	OSE + DEX	DEX 40 mg/d × 4d + OSE 75 mg bid × 10d	DEX	DEX 40 mg/d × 4d
Huang 2021	China	66/66	41/81	41/40	20/18.5	RA + DEX	RA 20 mg × 12w + DEX 40 mg/d × 4d	DEX	DEX 40 mg/d × 4d
An 2021	China	72/68	68/72	32.8	<30–50/<30–50	TAC + DEX	TA 0.03 mg/kg/d × 12w + DEX 40 mg × 4d	DEX	DEX 40 mg × 4d

C, control; DEX, dexamethasone; F, female; I, intervention; IVIG, intravenous gammaglobulin; M, male; mPRD, methylprednisolone; OSE, oseltamivir; PRD, prednisolone; RA; all-trans retinoic acid; rhTPO, recombinant human thrombopoietin; RTX, rituximab; TAC, tacrolimus; y, year.

The risk of bias is summarised in Appendix p 5. Most of these RCTs have a ‘high risk’ of blinding of participants and personnel and blinding of outcome assessment. Random sequence generation and allocation concealment were not sufficiently described in 7 studies (classified as ‘unclear’). All studies showed low risk of selective reporting. 3 RCTs were deemed at ‘high risk’ of incomplete outcome owing to lack of 6-month sustained response or grade 3/4 adverse events.

A total of 7 RCTs involving 854 patients and 5 regimens were available for direct comparison of combined therapeutic regimens (dexamethasone plus tacrolimus, all-trans retinoic acid, oseltamivir, rhTPO, or rituximab) *vs* dexamethasone treatment. The percentage of patients achieving early response was higher in all the combined group than in the dexamethasone group (79.7% *vs* 68.7%, OR = 1.82; 95% CI: 1.10–3.02) (I^2^ = 53.6%, *p* = 0.044) (Fig. 2B). The difference was more profound regarding 6-month sustained response, with 60.5% in the combined group and 37.4% in the dexamethasone group (OR = 2.57; 95% CI: 1.95–3.40) (I^2^ = 0.0%, *p* = 0.727) (Fig. 2B). Similar results were obtained when fixed models were used for early response (OR = 1.89; 95% CI: 1.36–2.62) and 6-month sustained response (OR = 2.58; 95% CI: 1.95–3.40), respectively (Appendix p 6).

**Fig. 3:**
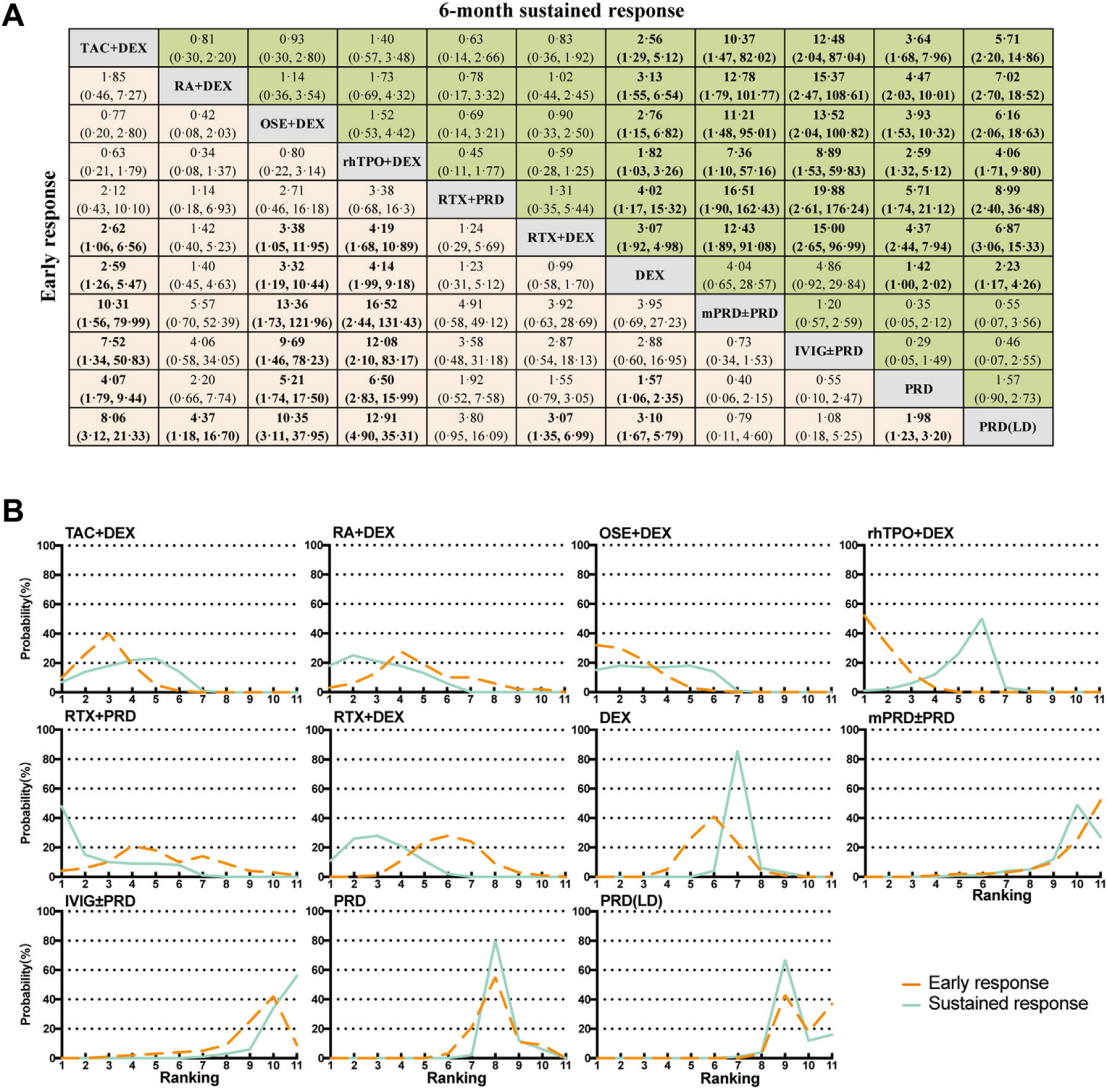
**Network meta-analysis of comparison of different treatments on early response and sustained response in newly-diagnosed adult primary immune thrombocytopenia**. (A) Pooled estimates of the network meta-analysis of early response and sustained response. Data in each cell is odds ratio (OR) (95% CrIs) for the comparison of row-defining treatment versus column-defining treatment. OR greater than 1 favor upper-row treatment. Significant results are highlighted in bold. (B) Profiles indicate the probability of each comparable treatment being ranked from first to last on early response and sustained response. DEX, dexamethasone; IVIG, intravenous gammaglobulin; mPRD, methylprednisolone; OSE, oseltamivir; PRD, prednisone; RA, all-trans retinoic acid; rhTPO, recombinant human thrombopoietin; RTX, rituximab; TAC, tacrolimus.

Dexamethasone and prednis(ol)one, the two reagents as the first choice for the newly diagnosed ITP, were compared using pairwise meta-analysis by pooling head-to-head data. In terms of early response, a total of 4 RCTs involving 505 patients were available for direct comparison. Pairwise meta-analysis showed that the percentage of patients achieving early response in the dexamethasone group was 67.2%, in comparison to 62.0% in the prednis(ol)one group. For sustained response, 6 RCTs were included involving 587 patients for pairwise meta-analysis. The response rate was 51.1% for dexamethasone and 43.5% for prednis(ol)one, but no significant statistical difference was reached in both early (OR = 1.71; 95% CI: 0.67–4.34) (I^2^ = 80.0%, *p* = 0.002) and sustained response (OR = 2.14; 95% CI: 0.94–4.86) (I^2^ = 75.9%, *p* = 0.001) using random model (Fig. 2C). Moreover, we also performed sensitive analysis using fixed model, and the results indicated that dexamethasone was slightly superior to prednis(ol)one in both early response and sustained response, consistent with the results derived from network meta-analysis (Fig. 3A and Appendix p 7).

Network meta-analysis of the early overall response within 4 weeks was performed in 16 studies (n = 1862). The definition of ‘early response’ is not consistent due to the long-time span of the included studies from 1985 to 2022, as shown in Appendix p 8. The result of early response is presented in the ladder diagram (Fig. 3). Compared with conventional monotherapy regimens, recently emerging regimens (rhTPO plus dexamethasone, tacrolimus plus dexamethasone, and oseltamivir plus dexamethasone) showed significantly better early response. rhTPO plus dexamethasone had a huge advantage in early response over dexamethasone or prednis(ol)one monotherapy (OR: 4.14, 95% CI: 1.99–9.18 versus dexamethasone; OR: 6.50, 95% CI: 2.83–15.99 versus prednis(ol)one). The Bayesian ranking probability analysis indicated that rhTPO plus dexamethasone was most likely ranked top of the list (SUCRA = 0.93), followed by oseltamivir plus dexamethasone (SUCRA = 0.87) and tacrolimus plus dexamethasone (SUCRA = 0.81) (Appendix p 10). The efficacy of rituximab plus dexamethasone was similar to the dexamethasone monotherapy (OR: 0.99, 95% CI: 0.58–1.70).

Besides, we also evaluated 6-month sustained response in 17 studies (n = 1875). The concrete definition of ‘sustained response’ is summarised in (Appendix p 9). The network meta-analysis indicated that combined regimens including tacrolimus plus dexamethasone, all-trans retinoic acid plus dexamethasone, oseltamivir plus dexamethasone, rhTPO plus dexamethasone, rituximab plus prednis(ol)one and rituximab plus dexamethasone showed superior sustained response compared with other monotherapy regimens. All-trans retinoic acid plus dexamethasone and rituximab plus prednis(ol)one showed obviously better sustained response rate than dexamethasone or prednis(ol)one monotherapy (OR: 3.13, 95% CI: 1.55–6.54, for all-trans retinoic acid plus dexamethasone *vs* dexamethasone; OR: 4.47, 95% CI: 2.03–10.01, for all-trans retinoic acid plus dexamethasone *vs* prednis(ol)one; OR: 4.02, 95% CI: 1.17–15.32, for rituximab plus prednis(ol)one *vs* dexamethasone; OR: 5.17, 95% CI: 1.74–21.12, for rituximab plus prednis(ol)one *vs* prednis(ol)one). The Bayesian ranking probability analysis indicated that rituximab plus prednis(ol)one was likely to be ranked first (SUCRA = 0.85), followed by rituximab plus dexamethasone (SUCRA = 0.80) and all-trans retinoic acid plus dexamethasone (SUCRA = 0.80) (Appendix p 10). Contrary to the early response, the efficacy of rhTPO plus dexamethasone achieved inferior sustained response as compared with other doublet regimens, although not significantly statistical differences.

Regarding the adverse events, the percentage of grade ≥3 AEs in 11 studies was recorded (Table 2). 15.3% of patients who received rituximab plus dexamethasone have grade ≥3 AEs. The incidence is much higher than other treatment regimens. All-trans retinoic acid plus dexamethasone and prednis(ol)one had similar percentage of grade ≥3 AEs (4.7% and 4.8%, respectively). As for conventional therapy, the incidence of grade ≥3 AEs of prednisolone (4.8%) is higher than dexamethasone (4.0%), which is consistent with previous studies.^22^, ^23^, ^24^ Oseltamivir plus dexamethasone and rhTPO plus dexamethasone had low incidence of grade ≥3 AEs (2.3% and 2.9%, respectively), while tacrolimus plus dexamethasone reported no incidence of grade ≥3 AEs. Almost all adverse events were manageable, while the therapy-related deaths were rare.

**Table 2:** Grade 3 or higher adverse events.

	OSE + DEX (n = 43)	RA + DEX (n = 64)	TAC + DEX (n = 48)	rhTPO + DEX (n = 102)	RTX + DEX (n = 111)	DEX (n = 729)	PRD (n = 248)
Total adverse events (grade 3–4)	1 (2.3%)	3 (4.7%)	0	3 (2.9%)	17(15.3%)	29 (4.0%)	12 (4.8%)
Anxiety or mood disorders	0	1 (1.6%)	0	0	0	1 (0.1%)	0
Dizziness	0	0	0	0	1 (0.9%)	1 (0.1%)	0
Oedema	1 (2.3%)	0	0	0	0	0	0
Fever	0	0	0	0	3 (2.7%)	2 (0.3%)	0
Gastrointestinal reactions	0	0	0	0	0	4 (0.5%)	2 (0.8%)
Heart palpitation	0	0	0	0	1 (0.9%)	0	0
Infection	0	0	0	0	0	0	1 (0.4%)
Insomnia	0	1 (1.6%)	0	0	0	2 (0.3%)	0
Dry skin	0	1 (1.6%)	0	0	0	0	0
Hypertension	0	0	0	0	0	1 (0.1%)	1 (0.4%)
Hyperglycaemia	0	0	0	0	0	8 (1.1%)	6 (2.4%)
Elevated liver enzymes or liver injury	0	0	0	0	1 (0.9%)	0	0
Intracranial hemorrhage	0	0	0	2 (2.0%)	0	0	0
Hypokalemia	0	0	0	0	0	1 (0.1%)	0
Thromboembolic event	0	0	0	1 (1.0%)	0	0	0
Hemorrhagic disorder	0	0	0	0	2 (1.8%)	3 (0.4%)	1 (0.4%)
Pneumonia	0	0	0	0	1 (0.9%)	0	0
Bleeding grade 3–4	0	0	0	0	2 (1.8%)	1 (0.1%)	0
Pain	0	0	0	0	1 (0.9%)	1 (0.1%)	0
Infusion-related anaphylaxis	0	0	0	0	1 (0.9%)	0	0
Delayed neutropenia	0	0	0	0	1 (0.9%)	0	0
Vasculitis	0	0	0	0	1 (0.9%)	0	0
Cataract	0	0	0	0	1 (0.9%)	0	0
Chest pain	0	0	0	0	0	1 (0.1%)	0
Restlessness	0	0	0	0	0	0	1 (0.4%)
Death	0	0	0	0	1 (0.9%)	3 (0.4%)	0

DEX, dexamethasone; OSE, oseltamivir; PRD, prednisolone; RA; all-trans retinoic acid; rhTPO, recombinant human thrombopoietin; RTX, rituximab; TAC, tacrolimus.

The comparison-adjusted funnel plot appears symmetric for early response, indicating the absence of small-study effects in the network (Appendix p 11). However, the comparison-adjusted funnel plot shows asymmetric for sustained response, providing an indication for the presence of small-study effects (Appendix p 12). The asymmetry was mainly due to studies comparing the response between prednis(ol)one and dexamethasone.

To test the robustness and reliability of the results, sensitive analysis of network meta-analysis was performed by excluding one study with around 30% of previously treated cases.^25^ The results are the same as the primary analysis (Appendix p 13).

## Discussion

The systematic review and network meta-analysis included 18 randomised controlled trials, assessing efficacy and safety of 11 therapeutic regimens in newly diagnosed ITP. Our study demonstrated that compared with dexamethasone or prednis(ol)one monotherapy, all the combination regimens containing corticosteroids achieved more satisfactory results in both early and sustained response. The rationale of the combined regimens is delicate, compensating corticosteroids with different mechanisms and/or with different time windows. Here, we concluded addition of oseltamivir, all-trans retinoic acid, rituximab, tacrolimus or rhTPO will indeed benefit the patients both in early and sustained responses.

The corticosteroids can protect platelet from clearance, prolong platelet survival, increase platelet production and decrease bleeding by a direct effect on blood vessels.^26^^,^^27^ Therefore, corticosteroids had been recommended as standard initial treatment for newly diagnosed ITP since 1996.^26^^,^^28^ The choice of either dexamethasone or prednis(ol)one has been debated for years. Previous studies showed that compared with prednis(ol)one, high-dose dexamethasone has a shorter time to response, fewer bleeding events and less adverse events, but has similar response at 6 months.^22^^,^^29^ However, a study by Xiao et al.^30^ reported no difference in adverse event or relapse rate between the two regimens, while dexamethasone tend to reach a higher sustained response. Our results are favorable to dexamethasone both in short-term and long-term response, as well as the incidence of grade ≥3 AEs, though they had no obvious statistical difference using pairwise meta-analysis. Additionally, dexamethasone was administered orally at 40 mg daily for 4 consecutive days, and prednis(ol)one at 1 mg/kg/d (maximum dose 80 mg) for 14–28 days. Dexamethasone may have a better treatment compliance in clinical practice. This network meta-analysis included more RCTs and larger sample size than previous studies, however, the number of studies and participants is still limited with high heterogeneity, which may not lead to significant difference. Nevertheless, neither dexamethasone nor prednis(ol)one can change the natural history of ITP and can only delay its progression.^31^

Relative insufficiency of TPO has been observed in patients with ITP.^32^ Thereafter, TPO-RAs were developed and licenced to treat ITP in US from 2008. Since then, they have been widely used among over 100 countries. rhTPO is a full-length glycosylated TPO, whose bind site is similar to the endogenous TPO.^33^^,^^34^ The median time to response of rhTPO is 4 days,^35^ shorter than other treatments and suitable for the emergency treatment of ITP.^34^^,^^36^ In our study, rhTPO plus dexamethasone showed the highest early response. We also expected similar short-term results combining with other TPO-RAs, such as eltrombopag,^16^ avatrombopag, and romiplostim, which are under investigation (NCT04346654). Nevertheless, the efficacy of rhTPO plus dexamethasone is not very prominent in sustained response, probably due to the short-term subcutaneous injection of rhTPO during hospitalization. However, it should not be ignored that all data including rhTPO are based on trials in Chinese ethic background, which may need further verification in a broader population.

The combination of rituximab plus corticosteroids showed a relatively low rate in terms of early response, equivalent to corticosteroids alone, because the median time to response of rituximab is around 4 weeks.^37^ However, this combination obtained the highest 6-month sustained response. It is of note that rituximab plus dexamethasone is evaluated in 3 RCTs, in which the cut-off value of platelet count for responders is higher than majority of other RCTs (50 × 10⁹/L instead of 30 × 10⁹/L), making the response rate lower than expected. Despite the slow onset, the effect of rituximab on B cells can last for several months. Furthermore, another meta-analysis showed that 12-month response of rituximab plus dexamethasone was much better than dexamethasone,^38^ suggesting the advantage of long-term remission of rituximab plus corticosteroids should be more profound. Meanwhile, we also noticed a significant higher incidence of grade 3 or higher adverse events, though all these were manageable. Rituximab had a longer follow-up period than the other regimens, which resulted in more adverse effects being recorded during the study period.

Adding other drugs, oseltamivir, all-trans retinoic acid or tacrolimus, also produced excellent sustained response, without increasing adverse events. These repurposed drugs are based on the advances in the pathogenesis of ITP. Oseltamivir is a sialidase inhibitor agent used to treat influenza in its on-label use, which can reduce the platelet uptake by hepatocytes in ITP.^39^ All-trans retinoic acid is known to treat acute promyelocytic leukemia and its immunoregulatory properties has been shown in ITP, namely restoring the balance of macrophages towards M2, regulating cytokines release and the proportion of T subgroup.^40^^,^^41^ Tacrolimus is a calcineurin inhibitor used for prophylaxis and treatment of graft-*vs*-host disease after organ transplantation and also used for refractory autoimmune diseases. Oral administration of these drugs is more convenient to deliver than some intravenous preparations like rituximab and subcutaneous injection of therapies like rhTPO. In a recent study (NCT03156452), addition of mycophenolate to glucocorticoid for the first line ITP treatment results in a higher cumulative response rate (78%, n = 46/59 *vs* 56%, n = 34/61) and reduces the risk of refractory or relapsed ITP, as compared with glucocorticoid alone.^18^ The primary endpoint of this study is treatment failure and initiation of second-line treatment, which did not meet the inclusion criteria and has not been included in our network meta-analysis. Nowadays, other new combined regimens, such as dexamethasone combined with sirolimus (ChiCTR1900020657), and glycyrrhetinic acid combined with dexamethasone (NCT03998982), are investigated underway. With these new therapies emerging, more detailed network meta-analysis can be done to obtain the optimal treatment for newly diagnosed ITP. In the future, more high-quality studies are expected.

The strength of this study is the inclusion of the most up-to-date published RCTs with median or high quality. The comprehensive network and pairwise meta-analyses indicated that corticosteroids-based combination therapy is superior to the conventional monotherapy. Besides, sensitive analyses were performed to ensure the reliability and robustness of the results.

However, this study has several limitations. First, newly emerging combination regimens (dexamethasone plus tacrolimus, all-trans retinoic acid, or oseltamivir) showed excellent response, but were evaluated in single trial with limited sample size. Subgroup analyses of demographic variables and source factors with high heterogeneity are difficult to carried out due to the small number of included studies and study participants. In addition, as most of the included RCTs had a ‘high risk’ of participant and personnel blinding and outcome assessment blinding, and ‘unclear’ random sequence generation and allocation concealment, which makes it impossible to perform sensitivity analysis after excluding these RCTs. Second, different cut-offs of platelet count to define early and sustained response as well as different time points to record early response may lead to biased results. Third, it remains unclear about the long-term effect of the evaluated regimens, as most of these RCTs were followed up for about 6–12 months. Fourth, non-English language trials were not included in this study, which may cause potential language bias and selection bias. Finally, the bleeding risk was not evaluated because of data incompleteness and different standards. High-quality head-to-head RCTs and real-world studies may provide answers to those questions.

ITP tend to present a chronic process in adults.^42^ Previous reports using questionnaire to evaluate the quality of life (QoL) among different diseases showed that patients with ITP, especially the chronic, had a worse QoL even below cancer patients.^43^^,^^44^ Therefore, it is worthwhile to investigate whether we should try to achieve a better remission in the early stage to delay the progression into chronic ITP.

In conclusion, this systematic review and network meta-analysis summarized all the feasible treatment regimens and demonstrated that combination regimens have more benefits than conventional corticosteroids monotherapy for newly diagnosed ITP. rhTPO plus dexamethasone showed the best early response, but its sustained response rate is relatively low. The addition of rituximab, oseltamivir, all-trans retinoic acid or tacrolimus to corticosteroids demonstrated the similar long-term efficacy, but the incidence of adverse events of rituximab plus dexamethasone is higher. Our study offers a guidance for the selection of the optimal regimen in the management of newly diagnosed ITP.

## Contributors

M.X. and M.H. contributed to the original concept and funded the research. Y.W. and L.S. contributed to data collection and analysis. The data were verified by M.X. and M.H. The manuscript was drafted by Y.W., L.S. and M.X. All authors provided major intellectual contributions to the manuscript, reviewed, and revised its content, and approved the final version. The corresponding authors had full access to all the data in the study and had final responsibility for the decision to submit for publication.

## Data sharing statement

The data that support the findings of this systematic review and network meta-analysis are shared upon request. The shared data included study-relevant data and statistical analysis plan. These data will be made available from the time of publication.

## Declaration of interests

We declare no competing interests.

## Appendix A Supplementary data

Caption for Appendix
