# Supplementary material for: Efficacy and safety of treatments in newly diagnosed adult primary immune thrombocytopenia: A systematic review and network meta-analysis
Source: eClinicalMedicine. 2022 Dec 14;56:101777. doi: 10.1016/j.eclinm.2022.101777 (PMC9791309; doi:10.1016/j.eclinm.2022.101777)
Supplement: Caption for Appendix [file mmc1.docx]

Appendix

Checklist of the PRISMA extension for network meta-analysis
