# Supplementary material for: Efficacy and safety of treatments in newly diagnosed adult primary immune thrombocytopenia: A systematic review and network meta-analysis
Source: eClinicalMedicine. 2022 Dec 14;56:101777. doi: 10.1016/j.eclinm.2022.101777 (PMC9791309; doi:10.1016/j.eclinm.2022.101777)
Supplement: Appendix [file mmc2.pdf]

Supplementary appendix

Supplement to:
Efficacy and safety of treatments in newly diagnosed adult primary immune thrombocytopenia: A systematic review and network meta-analysis

Appendix **Page**

1.	Literature search strategy	1-2
2.	Convergence of the four chains established by inspection of the Brooks-Gelman-Rubin diagnostic and the trace plot	3-4
3.	Summary of bias risk assessment of included studies using the Cochrane risk of bias tool	5
4.	Pairwise meta-analysis of comparison of dexamethasone-containing doublet versus dexamethasone in patients with newly-diagnosed primary immune thrombocytopenia using fixed model	6
5.	Pairwise meta-analysis of comparison of dexamethasone versus prednisone in patients with newly-diagnosed primary immune thrombocytopenia using fixed model	7
6.	Definition of ER, and number of patients in total and those achieving ER in each study	8
7.	Definition of SR, and number of patients in total and those achieving SR in each study	9
8.	Bayesian ranking profiles of comparable treatments on sustained response and early response for patients with newly-diagnosed primary immune thrombocytopenia	10
9.	Comparison-adjusted funnel plot for the early response network	11
10.	Comparison-adjusted funnel plot for the sustained response network	12
11.	Sensitive analysis of network meta-analysis on early response and sustained response by excluding one study with around 30% of previously treated cases	13

Appendix 1. Literature search strategy

1.1 Search strategy on PubMed

((((((((((((((((((((((((Purpura, Thrombocytopenic, Idiopathic[Title]) OR (Idiopathic Thrombocytopenic Purpura[Title])) OR (Idiopathic Thrombocytopenic Purpuras[Title])) OR (Purpura, Idiopathic Thrombocytopenic[Title])) OR (Purpuras, Idiopathic Thrombocytopenic[Title])) OR (Thrombocytopenic Purpura, Idiopathic[Title])) OR (Thrombocytopenic Purpuras, Idiopathic[Title])) OR (Immune Thrombocytopenic Purpura[Title])) OR (Immune Thrombocytopenic Purpuras[Title])) OR (Purpura, Immune Thrombocytopenic[Title])) OR (Purpuras, Immune Thrombocytopenic[Title])) OR (Thrombocytopenic Purpura, Immune[Title])) OR (Thrombocytopenic Purpuras, Immune[Title])) OR (Immune Thrombocytopenia[Title])) OR (Immune Thrombocytopenias[Title])) OR (Thrombocytopenia, Immune[Title])) OR (Thrombocytopenias, Immune[Title])) OR (Thrombocytopenic Purpura, Autoimmune[Title])) OR (Autoimmune Thrombocytopenia[Title])) OR (Autoimmune Thrombocytopenias[Title])) OR (Thrombocytopenia, Autoimmune[Title])) OR (Thrombocytopenias, Autoimmune[Title])) OR (Autoimmune Thrombocytopenic Purpura[Title])) OR (Autoimmune Thrombocytopenic Purpuras[Title])) OR (Purpura, Autoimmune Thrombocytopenic[Title])) OR (Purpuras, Autoimmune Thrombocytopenic[Title])) OR (Purpura, Thrombocytopenic, Autoimmune[Title]) **Filters:** Randomized Controlled Trial

1.2 Search strategy on Embase

#1 ('autoimmune thrombocytopeni*':ti OR 'idiopathic thrombocytopenic purpura':ti OR itp:ti OR 'immune thrombocytopenia':ti OR 'primary thrombocytopeni*':ti) NOT relapse*:ti NOT refractory:ti NOT chronic:ti NOT second:ti
#2 'randomized controlled trial'/de
#3 #1 AND #2

1.3 Search strategy on Cochrane Central Register of Controlled Trials

"immune thrombocytopenia":ti,ab,kw OR "primary thrombocytopeni*":ti,ab,kw OR autoimmune thrombocytopenia:ti,ab,kw OR ITP:ti,ab,kw NOT chronic:ti (Word variations have been searched)

1.4 Search strategy on ClinicalTrials.gov databases

Completed Studies | Interventional Studies | immune thrombocytopenia | Adult
Also searched for Idiopathic thrombocytopenic purpura and Immune thrombocytopenic purpura.

1.5 Excluded studies with reasons for exclusion at full-text screening stage

Reference	Reason for exclusion
PRAITUAN 2009[1]	No relevant outcomes reported
Donald 2012[2]	Incorrect population
GU 2013[3]	No relevant outcomes reported
Nyein 2019[4]	No relevant outcomes reported
Matzdorff 2020[5]	No relevant outcomes
Charlotte 2021[6]	No relevant outcomes reported

Reference

1. Praituan W, Rojnuckarin P. Faster platelet recovery by high-dose dexamethasone compared with standard-dose prednisolone in adult immune thrombocytopenia: A prospective randomized trial. J Thromb Haemost. 2009;7(6):1036–8.
2. Arnold DM, Heddle NM, Carruthers J, Cook DJ, Crowther MA, Meyer RM, et al. A pilot randomized trial of adjuvant rituximab or placebo for nonsplenectomized patients with immune thrombocytopenia. Blood. 2012 Feb;119(6):1356–62.
3. GU S. A clinical comparative study on treatment of severe newly diagnosed immune thrombocytopenia by recombinant human thrombopoietin combined with glucocorticoid．. China Med Abstr Intern Med. 2013;34(10):228–9.
4. Nyein H. High Dose Dexamethasone vs Prednisolone Therapy in Newly Diagnosed Myanmar Adult Patients with Primary Immune Thrombocytopenia. Res Pract Thromb Haemost. 2019;3(1):633–4.
5. Axel Matzdorff, MDPhD, Mascha Binder, MD, Falk Nimmerjahn, PhD, Oliver Meyer MP. A Phase II Study to Investigate the Efficacy and Safety of Eltrombopag in Combination with Dexamethasone As First-Line Treatment in Adult Patients with Newly Diagnosed Primary ITP (XPAG-ITP). Blood [Internet]. 2020;136(Supplement 1):36–7. Available from: http://dx.doi.org/10.1182/blood-2020-134318
6. Bradbury CA, Pell J, Hill Q, Bagot C, Cooper N, Ingram J, et al. Mycophenolate Mofetil for First-Line Treatment of Immune Thrombocytopenia. N Engl J Med. 2021;385(10):885–95.

Appendix 2. Convergence of the four chains established by inspection of the Brooks-Gelman-Rubin diagnostic and the trace plot

2.1 Brooks-Gelman-Rubin diagnostic for early response

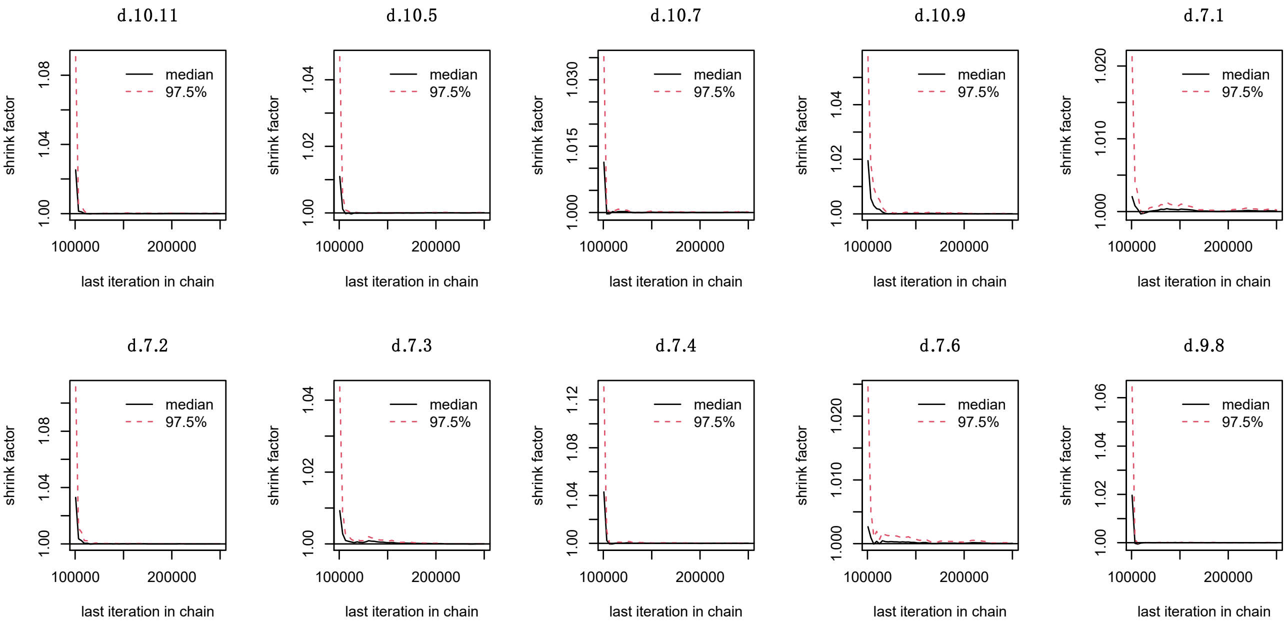

2.2 Trace plot for early response

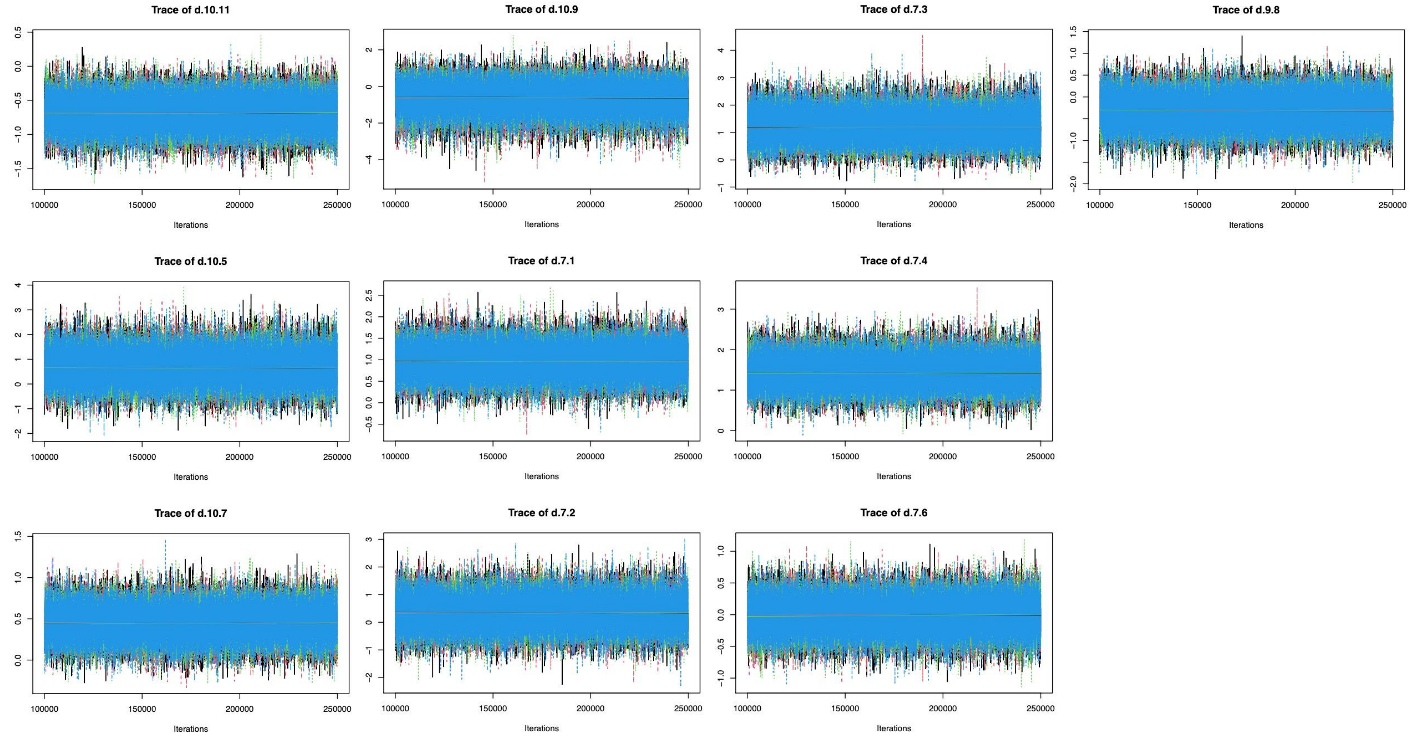

2.3 Brooks-Gelman-Rubin diagnostic for 6-month sustained response

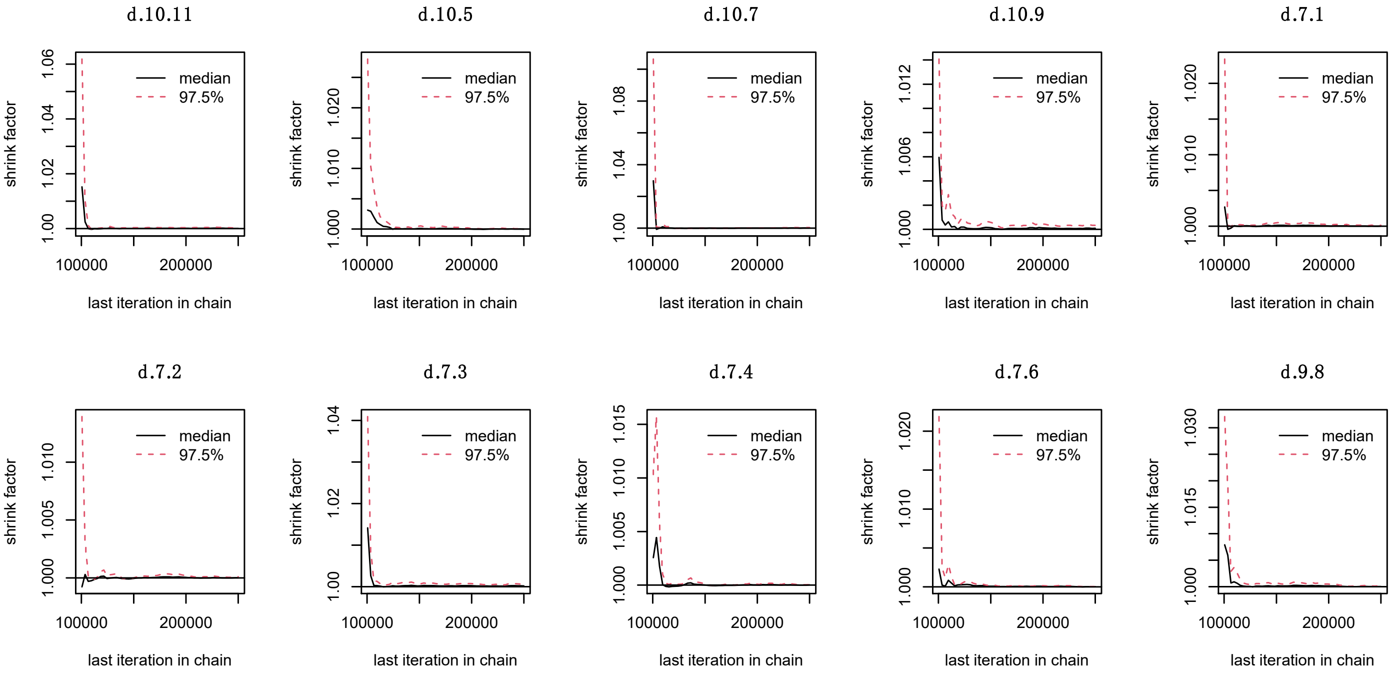

2.4 Trace plot for 6-month sustained response

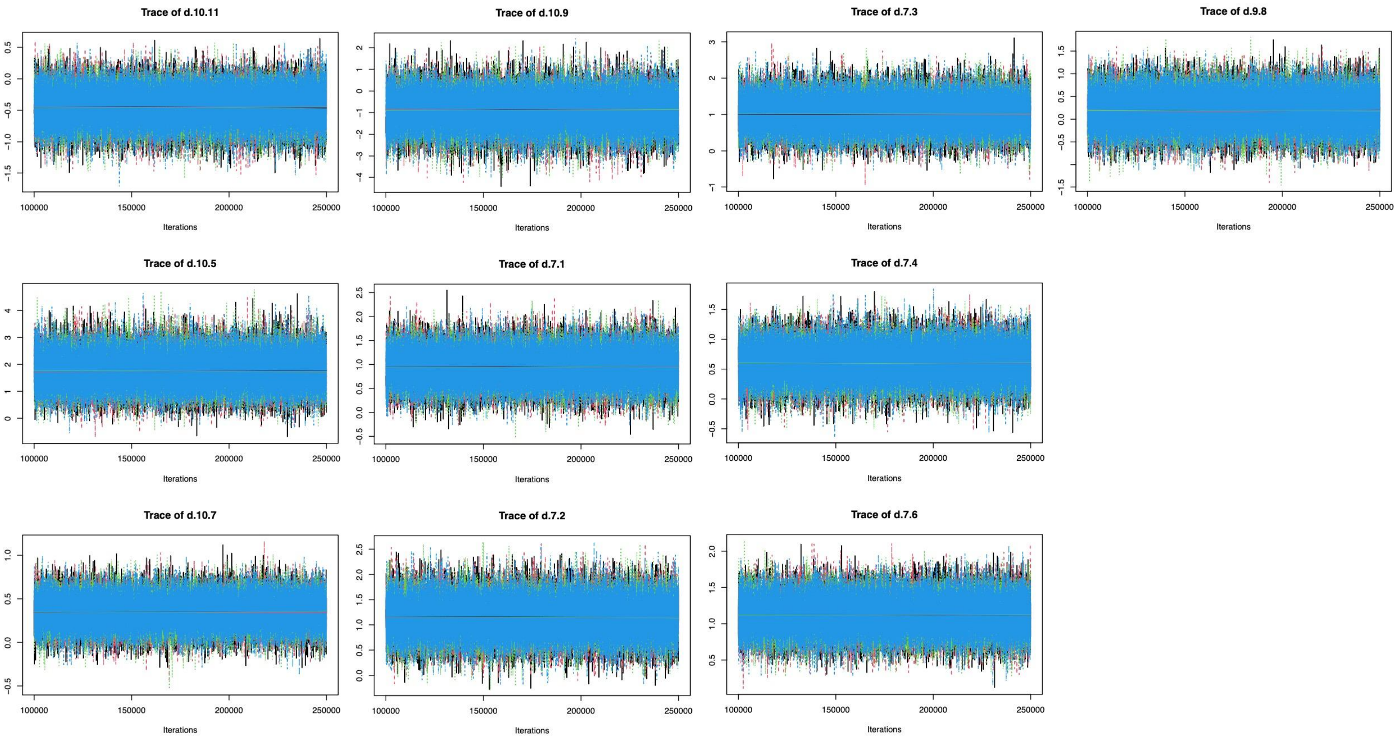

Appendix 3. Summary of bias risk assessment of included studies using the Cochrane risk of bias tool

Study ID	Random sequence generation	Allocation concealment	Blinding of participants and personnel	Blinding of outcome assessment	Incomplete outcome data	Selective reporting
An 2021	-	-	+	+	-	-
Huang 2021	-	-	+	-	-	-
Sun 2021	-	-	+	-	-	-
Sadeghi 2020	-	-	+	+	-	-
Yu 2020	-	-	+	+	-	-
Datta 2018	?	?	+	+	+	
Matschke 2016	?	?	+	+	+	-
Wei 2016	-	-	+	+	-	-
Din 2015	?	?	+	+	-	-
Gudbrandsdottir 2013	-	-	+	+	-	-
Mashhadi 2012	?	?	+	+	-	-
Li 2011	?	?	+	+	-	-
Bae 2010	?	?	+	+	+	-
Zaja 2010	?	?	+	+	-	-
Godeau 2002	-	-	+	+	-	-
Jacobs 1994	-	?	+	+	+	-
Bellucci 1988	-	-	+	+	-	-
Mazzucconi 1985	?	?	+	+	-	-

Appendix 4. Pairwise meta-analysis of comparison of dexamethasone-containing doublet versus dexamethasone in patients with newly-diagnosed primary immune thrombocytopenia using fixed model

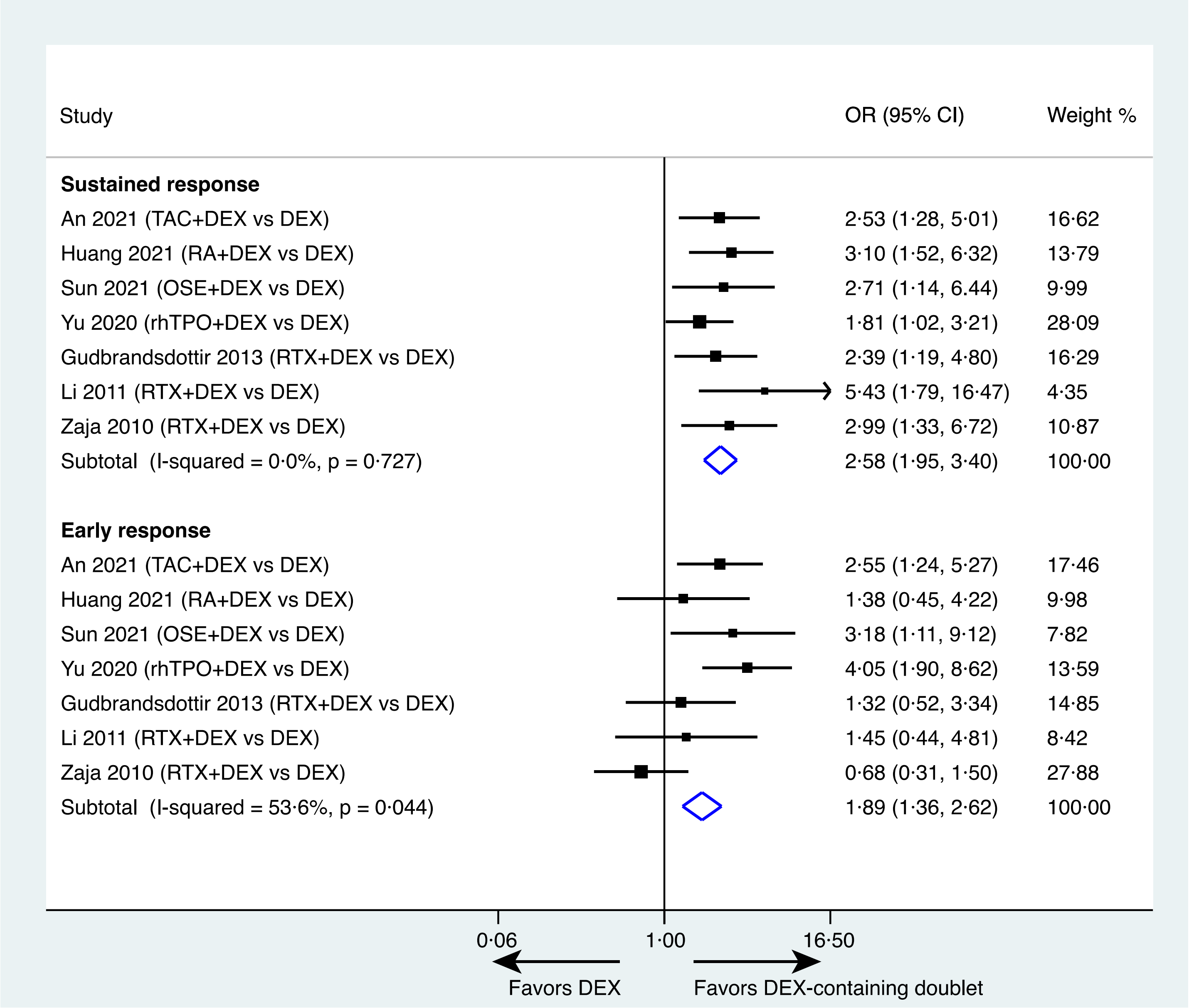

OR, odds ratio; CI, confidence interval; TAC, tacrolimus; DEX, dexamethasone; RA; all-trans retinoic acid; OSE, oseltamivir; rhTPO, recombinant human thrombopoietin; RTX, rituximab.

Appendix 5. Pairwise meta-analysis of comparison of dexamethasone versus prednisone in patients with newly-diagnosed primary immune thrombocytopenia using fixed model

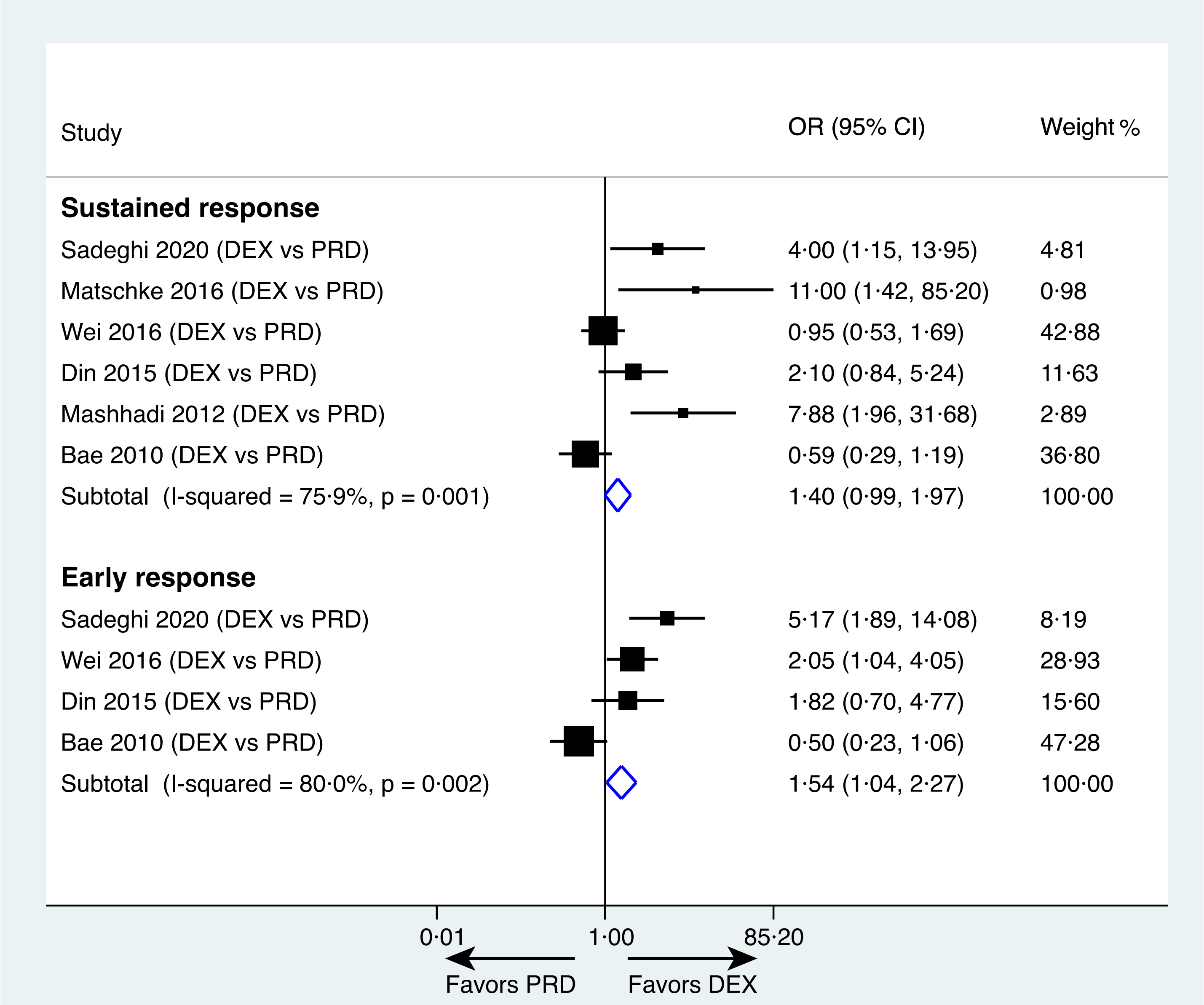

OR, odds ratio; CI, confidence interval; DEX, dexamethasone; PRD, prednisone

Appendix 6. Definition of ER, and number of patients in total and those achieving ER in each study

Study ID	Definition of ER, PLT (*10^9)	TAC+ DEX (n/N)	RA+ DEX (n/N)	OSE+ DEX (n/N)	rhTPO +DEX (n/N)	RTX+ PRD (n/N)	RTX+ DEX (n/N)	DEX (n/N)	mPRD ±PRD (n/N)	IVIG± PRD (n/N)	PRD (n/N)	PRD (LD) (n/N)
Mazzucconi 1985	60										21/32	24/37
Bellucci 1988	30										65/112	41/111
Jacobs 1994	50									19/26	14/17	
Godeau 2002	50								33/60	35/56		
Bae 2010	30							52/76			61/75	
Zaja 2010	50						18/49	24/52				
Li 2011	50						25/31	23/31				
Gudbrandsdotir 2013	50						53/62	58/71				
Din 2015	30							25/61			8/29	
Wei 2016	30							78/95			67/97	
Datta 2018	NA					21/26					18/26	
Sadeghi 2020	30							25/36			11/36	
Yu 2020	30				89/100			64/96				
Sun 2021	30			37/43				31/47				
Huang 2021	30		60/66					58/66				
An 2021	50	55/72						38/68				

ER, early response; TAC, tacrolimus; DEX, dexamethasone; RA, all-trans retinoic acid; OSE, oseltamivir; rhTPO, recombinant human thrombopoietin; RTX, rituximab; mPRD, methylprednisolone; IVIG, intravenous gammaglobulin; PRD (LD), low-dose PRD; n, number of patients had ER; N, total number of patients in each treatment; NA, not applicable.

Appendix 7. Definition of SR, and number of patients in total and those achieving SR in each study

Study ID	Definition of SR, PLT (*10^9)	TAC+ DEX (n/N)	RA+ DEX (n/N)	OSE+ DEX (n/N)	rhTPO+ DEX (n/N)	RTX+ PRD (n/N)	RTX+ DEX (n/N)	DEX (n/N)	mPRD± PRD (n/N)	IVIG± PRD (n/N)	PRD (n/N)	PRD (LD) (n/N)
Bellucci 1988	100										48/112	36/111
Jacobs 1994	100									3/26	5/17	
Godeau 2002	50								24/60	20/56		
Bae 2010	30							19/76			27/75	
Zaja 2010	50						31/49	19/52				
Li 2011	50						24/31	12/31				
Mashhadi 2012	30							27/30			16/30	
Gudbrandsdotir 2013	50						35/62	25/71				
Din 2015	30							32/61			10/29	
Wei 2016	30							38/95			40/97	
Matschke 2016	50							11/13			3/9	
Datta 2018	NA					20/26					10/26	
Sadeghi 2020	30							32/36			24/36	
Yu 2020	30				51/100			35/96				
Sun 2021	30			23/43				14/47				
Huang 2021	30		45/66					27/66				
An 2021	50	47/72						29/68				

SR, sustained response; TAC, tacrolimus; DEX, dexamethasone; RA, all-trans retinoic acid; OSE, oseltamivir; rhTPO, recombinant human thrombopoietin; RTX, rituximab; mPRD, methylprednisolone; IVIG, intravenous gammaglobulin; PRD (LD), low-dose PRD; n, number of patients had SR; N, total number of patients in each treatment; NA, not applicable.

Appendix 8. Bayesian ranking profiles of comparable treatments on sustained response and early response for patients with newly-diagnosed primary immune thrombocytopenia

	Primary analysis		Sensitivity analysis	
	SR	ER	SR	ER
RTX+PRD	0.85	0.57	0.86	0.57
RA+DEX	0.80	0.61	0.81	0.62
RTX+DEX	0.80	0.49	0.75	0.46
OSE+DEX	0.75	0.87	0.76	0.87
TAC+DEX	0.72	0.81	0.73	0.81
rhTPO+DEX	0.57	0.93	0.58	0.93
DEX	0.39	0.50	0.39	0.51
PRD	0.28	0.30	0.28	0.31
PRD(LD)	0.16	0.11	0.16	0.11
mPRD±PRD	0.12	0.10	0.12	0.10
IVIG±PRD	0.06	0.20	0.06	0.20

Number (percentage of surface under the cumulative ranking curve (SUCRA)) in each cell indicates the probability of each treatment being ranked from first (high value) to last (low value) on sustained response and early response in primary and sensitivity analysis. SR, sustained response; ER, early response; TAC, tacrolimus; DEX, dexamethasone; RA, all-trans retinoic acid; OSE, oseltamivir; rhTPO, recombinant human thrombopoietin; RTX, rituximab; mPRD, methylprednisolone; IVIG, intravenous gammaglobulin; PRD (LD), low-dose PRD.

Appendix 9. Comparison-adjusted funnel plot for the early response network

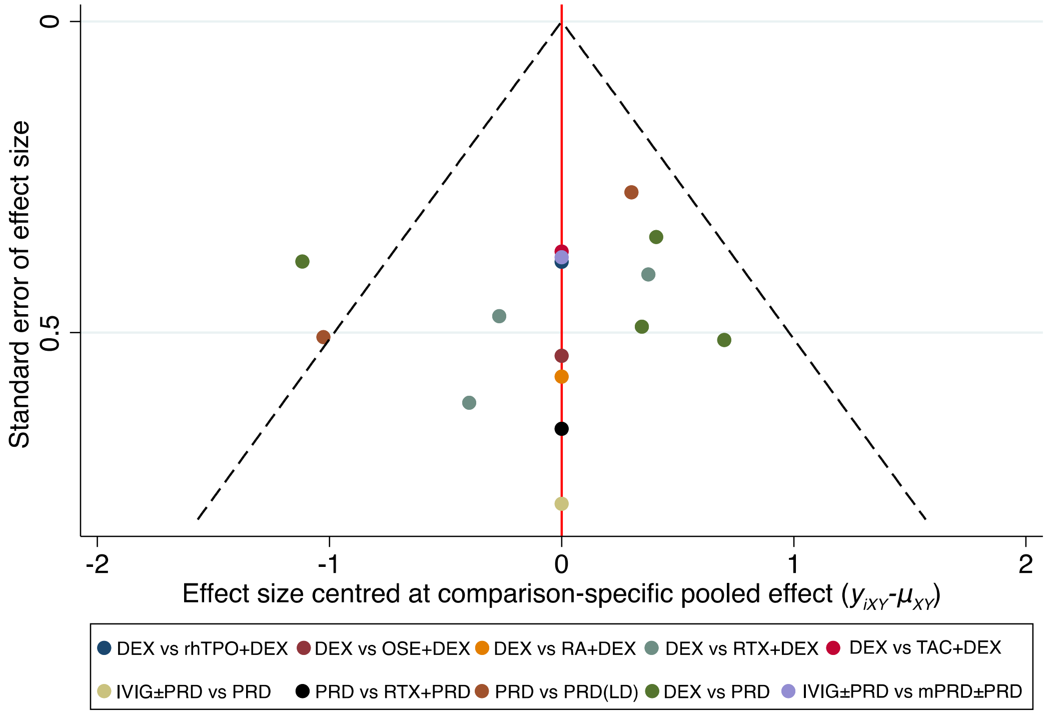

Appendix 10. Comparison-adjusted funnel plot for the sustained response network

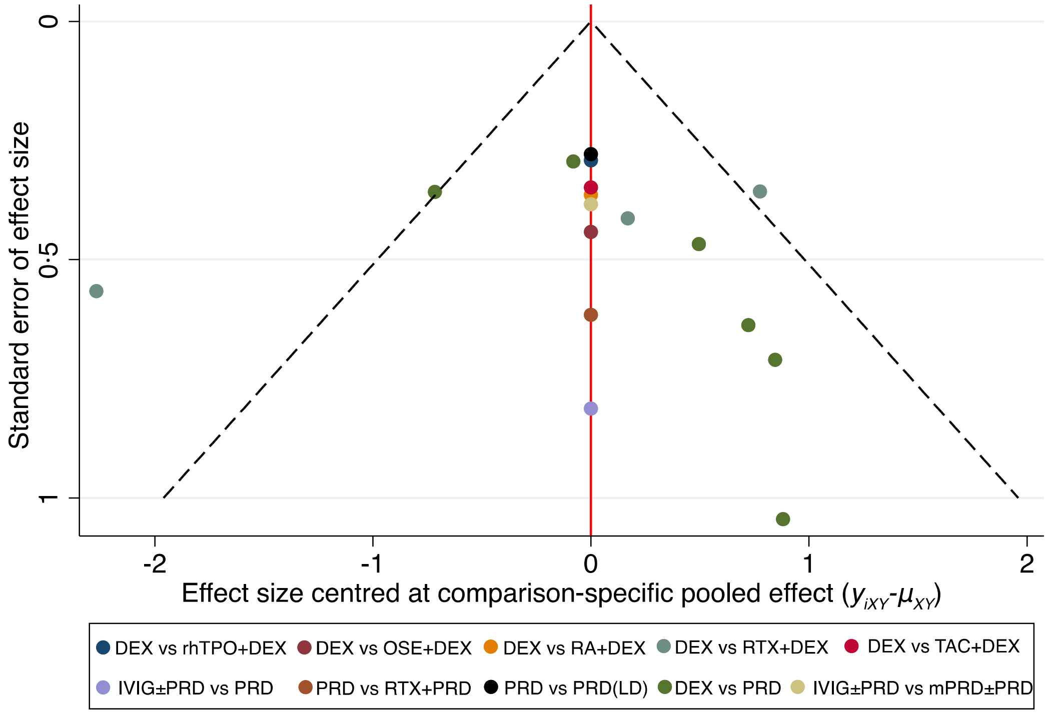

Appendix 11. Sensitive analysis of network meta-analysis on early response and sustained response by excluding one study with around 30% of previously treated cases

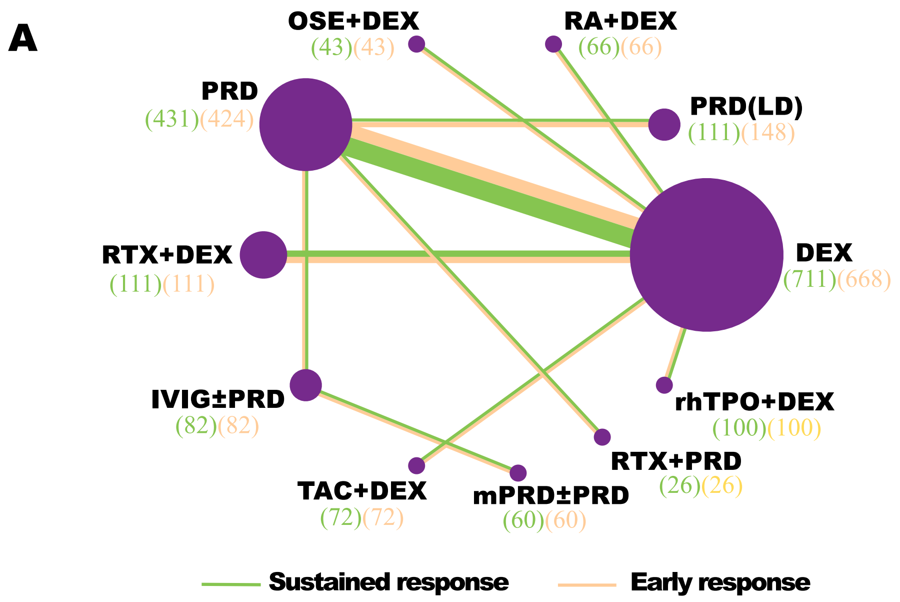

B

6-month sustained response (upper right); Early response (lower left)

**TAC+DEX**	0.81 (0.30, 2.20)	0.92 (0.30, 2.78)	1.41 (0.57, 3.46)	0.64 (0.14, 2.66)	0.96 (0.40, 2.30)	**2.56 (1.29, 5.13)**	**10.38 (1.49, 84.95)**	**12.49 (2.10, 88.19)**	**3.64 (1.69, 7.90)**	**5.72 (2.21, 14.83)**
1.85 (0.46, 7.17)	**RA+DEX**	1.14 (0.36, 3.51)	1.73 (0.69, 4.40)	0.79 (0.17, 3.36)	1.19 (0.49, 2.91)	**3.16 (1.55, 6.59)**	**12.79 (1.83, 106.48)**	**15.35 (2.59, 110.14)**	**4.49 (2.04, 10.11)**	**7.03 (2.69, 18.84)**
0.78 (0.20, 2.80)	0.42 (0.09, 2.03)	**OSE+DEX**	1.52 (0.54, 4.37)	0.69 (0.14, 3.20)	1.04 (0.38, 2.96)	**2.77 (1.17, 6.82)**	**11.27 (1.49, 99.59)**	**13.53 (2.04, 103.46)**	**3.94 (1.55, 10.31)**	**6.18 (2.11, 18.72)**
0.62 (0.21, 1.80)	0.34 (0.08, 1.36)	0.80 (0.22, 3.13)	**rhTPO+DEX**	0.46 (0.11, 1.78)	0.68 (0.31, 1.50)	**1.82 (1.03, 3.25)**	**7.40 (1.10, 58.48)**	**8.86 (1.53, 59.97)**	**2.59 (1.32, 5.13)**	**4.07 (1.71, 9.77)**
2.10 (0.42, 9.88)	1.14 (0.18, 6.82)	2.72 (0.46, 15.75)	3.39 (0.67, 16.26)	**RTX+PRD**	1.51 (0.39, 6.34)	**4.01 (1.15, 15.28)**	**16.44 (1.90, 168.11)**	**19.71 (2.59, 177.29)**	**5.69 (1.73, 20.87)**	**8.97 (2.39, 36.46)**
**2.88 (1.12, 7.51)**	1.56 (0.43, 5.91)	**3.71 (1.13, 13.55)**	**4.62 (1.78, 12.60)**	1.38 (0.31, 6.46)	**RTX+DEX**	2.66 (1.58, 4.55)	**10.80 (1.65, 83.59)**	**12.95 (2.30, 86.30)**	**3.79 (2.01, 7.16)**	**5.94 (2.58, 13.93)**
**2.59 (1.26, 5.51)**	1.40 (0.45, 4.61)	**3.33 (1.20, 10.53)**	**4.15 (1.99, 9.25)**	1.23 (0.32, 5.17)	0.90 (0.49, 1.65)	**DEX**	4.04 (0.66, 29.41)	4.85 (0.93, 30.10)	**1.42 (1.00, 2.02)**	**2.23 (1.16, 4.31)**
**10.32 (1.56, 80.68)**	5.60 (0.70, 52.83)	**13.31 (1.77, 124.70)**	**16.55 (2.48, 132.10)**	4.93 (0.59, 49.35)	3.58 (0.57, 27.00)	3.96 (0.70, 27.22)	**mPRD±PRD**	1.20 (0.56, 2.58)	0.35 (0.05, 2.08)	0.55 (0.07, 3.56)
**7.47 (1.33, 51.12)**	4.07 (0.59, 33.88)	**9.70 (1.48, 78.99)**	**12.04 (2.11, 83.42)**	3.57 (0.49, 31.92)	2.59 (0.49, 16.94)	2.86 (0.61, 17.22)	0.73 (0.34, 1.52)	**IVIG±PRD**	0.29 (0.05, 1.46)	0.46 (0.07, 2.53)
**4.05 (1.78, 9.53)**	2.19 (0.66, 7.66)	**5.23 (1.73, 17.51)**	**6.51 (2.80, 15.90)**	1.92 (0.53, 7.63)	1.41 (0.68, 2.93)	**1.56 (1.06, 2.34)**	0.40 (0.06, 2.12)	0.55 (0.09, 2.45)	**PRD**	1.57 (0.91, 2.72)
**8.01 (3.10, 21.29)**	**4.34 (1.19, 16.63)**	**10.37 (3.10, 37.95)**	**12.90 (4.92, 35.26)**	3.82 (0.96, 16.32)	**2.79 (1.17, 6.69)**	**3.10 (1.67, 5.78)**	0.78 (0.11, 4.52)	1.08 (0.18, 5.24)	**1.98 (1.23, 3.19)**	**PRD(LD)**

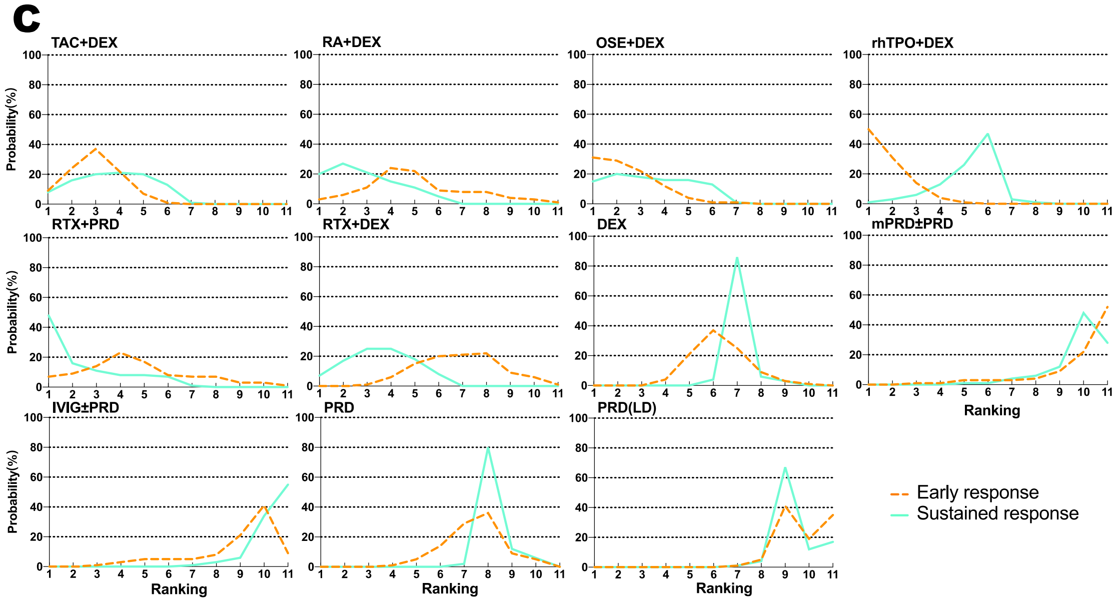

(A) Network diagrams of comparisons on early response (ER) and sustained response (SR). (B) Pooled estimates of the network meta-analysis of ER and SR. Data in each cell is odds ratio (OR) (95% CrIs) for the comparison of row-defining treatment versus column-defining treatment. OR greater than 1 favor upper-row treatment. Significant results are highlighted in bold. (C) Profiles indicate the probability of each comparable treatment being ranked from first to last on early response and sustained response. PRD, prednisone; IVIG, intravenous gammaglobulin; mPRD, methylprednisolone; DEX, dexamethasone; RTX, rituximab; rhTPO, recombinant human thrombopoietin; OSE, oseltamivir; RA; all-trans retinoic acid; TAC, tacrolimus
